# Behaviour of Hybrid Steel and FRP-Reinforced Concrete—ECC Composite Columns under Reversed Cyclic Loading

**DOI:** 10.3390/s18124231

**Published:** 2018-12-02

**Authors:** Fang Yuan, Liping Chen, Mengcheng Chen, Kaicheng Xu

**Affiliations:** School of Civil Engineering and Architecture, East China Jiaotong University, Nanchang 330013, China; zlh9508@163.com (L.C.); xkcxj@163.com (K.X.)

**Keywords:** engineered cementitious composite (ECC), hybrid reinforced, composite column, fibre-reinforced polymer, experiment, cyclic load, ductility

## Abstract

Fibre-reinforced polymer (FRP) is used widely in concrete structures owing to its noncorrosive, light-weight, nonmagnetic, and high tensile-strength properties. However, the FRP-reinforced concrete flexural member exhibits low ductility owing to the linear–elastic property of FRP reinforcement. Hybrid steel—FRP-reinforced concrete members exhibit good strength and ductility under flexure owing to the inelastic deformation of steel reinforcement. The existing investigations have focused on the mechanical behaviours of the hybrid steel—FRP-reinforced flexural members. Only few studies have been reported on the members under combined flexural and compression loads, such as columns, owing to the poor compressive behaviour of FRP bars. We herein propose a new type of hybrid steel—FRP-reinforced concrete—engineered cementitious composite (ECC) composite column with ECC applied to the plastic hinge region and tested it under reversed cyclic loading. The hybrid steel—FRP-reinforced concrete column was also tested for comparison. The influence of matrix type in the plastic hinge region on the failure mode, crack pattern, ultimate strength, ductility, and energy dissipation capacity, of the columns were evaluated systematically. We found that the substitution of concrete with ECC in the plastic hinge zone can prevent the local buckling of FRP bars efficiently, and subsequently improve the strength and ductility of the column substantially.

## 1. Introduction

The corrosion of reinforcing steel is a significant problem for reinforced concrete (RC) structures subjected to aggressive environments, such as marine structures, bridges, and parking garages. The corrosion process could cause the loss of serviceability or even the failure of load-carrying capacity. Composite materials made of fibres embedded in a polymeric resin, also known as fibre-reinforced polymer (FRP), is an alternative to steel reinforcement in RC structures, because of its noncorrosive property [1,2,3,4,5,6,7,8,9,10,11,12]. Furthermore, FRP materials exhibit remarkable properties of being nonmagnetic, high in tensile strength, and light weight, which render them suitable for applications in structural engineering [2,4]. However, FRP rebars exhibit linear elastic property up to failure, thus resulting in the brittle structural behaviour of FRP-reinforced concrete structures. Hence, FRP-reinforced concrete members must be designed to achieve the concrete compression failure mode other than FRP reinforcement rupture [13,14,15]. However, the inelastic deformation by concrete crushing prior to failure is still limited.

The combination layout of steel and FRP reinforcement can improve the mechanical behaviour of concrete flexural members. A large number of experiments have been conducted on hybrid steel–FRP-reinforced concrete beams [16,17,18,19,20,21,22,23,24,25]. Many have agreed that the ductility of beams reinforced with hybrid beams can be improved significantly through the substantial inelastic deformation of steel reinforcement compared with those reinforced with FRP alone. Recently, Yuan and Chen [26] studied the plastic hinge regions in hybrid steel–FRP-reinforced concrete beams through the finite element method. The existing investigations on hybrid-reinforced members concentrated on flexural members; few studies have focused on the members under combined flexural and compression loads, such as columns. The low compression strength of FRP reinforcement limits its application in compression members, which is only 30%—60% of its tensile strength. However, the concrete cover is likely to spall under reversed cyclic loading. In this case, the FRP bars’ lateral loss confinement reduces their compression strength further, and finally causes premature failure.

High-performance fibre-reinforced cement-based composites (HPFRCCs) can effectively improve the safety and integrity of structures. Among them, engineered cementitious composite (ECC) is an important representative. ECC is a new building material with ultrahigh tensile ductility and excellent crack control ability. Under uniaxial tension, the tensile strain capacity can reach 3% with a crack spacing of 3—5 mm, and a crack width of approximately 60 μm [27,28]. Under compression, the strain at peak stress is approximately two times that of concrete, thereby benefitting the ductility of the structural members that failed in the matrix compression [29]. Yuan et al. [30] studied the behaviour of steel-reinforced ECC column under eccentric compression and found that the steel-reinforced ECC columns exhibit higher ductility than the RC columns. Furthermore, evident ECC spalling was not observed during the reversed cyclic loading tests [31,32,33]. With these unique characteristics, ECC is expected to improve the integrity, ductility, and durability of hybrid steel–FRP-reinforced columns.

Recently, Ge et al. [34] investigated the flexural behaviour of ECC–concrete hybrid composite beams reinforced with FRP bars and steel bars. Test results showed that cracking, yield, ultimate moments, and the stiffness of hybrid reinforced concrete and ECC beams are improved compared with traditional concrete beams with the same reinforcement, due to the excellent tensile properties of ECC materials. It was still focused on flexural members other than the members under combined flexural and compression loads. This paper is an attempt to investigate the seismic behavior of hybrid steel–FRP-reinforced concrete–ECC composite column which has not been previously investigated. ECCs are applied only to the plastic hinge region of the column; they are expected to improve the performance–cost ratio and guarantee the good seismic performance of the composite column simultaneously. The mechanical behaviour of the hybrid reinforced concrete–ECC composite column was tested and compared with that of the hybrid reinforced concrete column. The influence of matrix type in the plastic hinge region on the failure model, crack pattern, ultimate strength, ductility, and energy dissipation capacity, of the columns are evaluated systematically.

## 2. Design Philosophy

**Figure 1:**
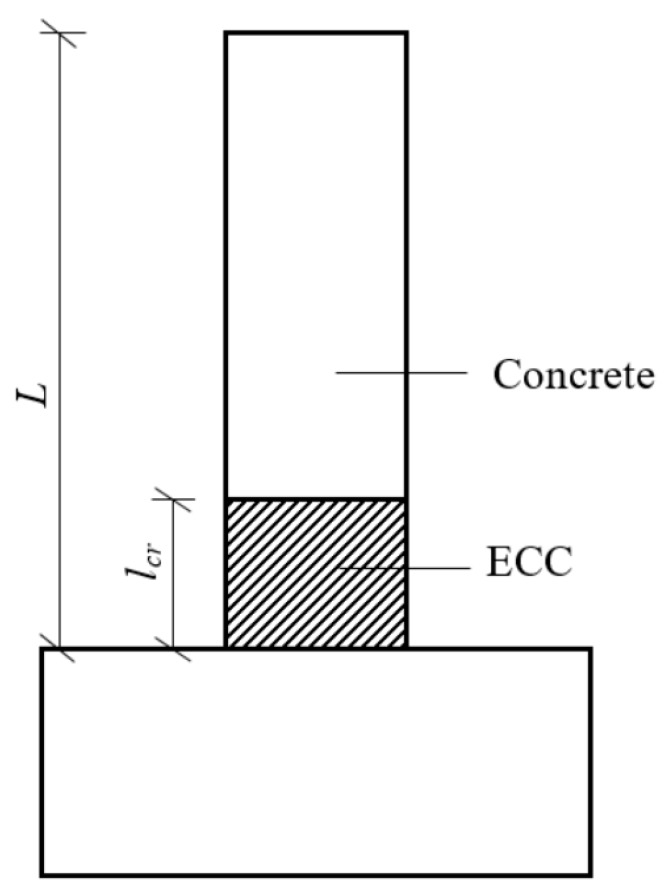
Schematic of ECC use area for concrete–ECC composite columns.

Owing to the higher cost of ECCs than that of concrete, it is not economical to use ECCs throughout the column height. For the concrete–ECC composite column, ECCs should be used strategically in the critical stress area, to fully exploit the superior deformation ability of ECCs to improve the seismic performance of the member. Hence, the concrete–ECC composite column uses only the ECC near the column base, and conventional concrete is used in other areas, as shown in Figure 1. To utilise the ECC, the plastic hinge must be ensured to first appear in the ECC area other than the concrete area. Otherwise, the composite column will fail at the concrete segment, thus resulting in the meaningless use of the ECC. Based on this design criterion, the critical ECC use area can be determined based on the following equations:

(1) $$\frac{M_{y1}}{L-l_{cr}}=\frac{M_{y2}}{L}$$

(2) $$l_{cr}=\frac{\left(M_{y2}-M_{y1}\right)L}{M_{y2}}$$

where *l_cr_* is the critical ECC pouring height, *L* is the column height, *M_y_*_1_ is the yield moment of the hybrid reinforced concrete section, and *M_y_*_2_ is the yield moment of the hybrid reinforced ECC section. Provided that the ECC pouring height is slightly greater than the critical height *l_cr_*, the plastic hinge zone and final failure region will occur in the ECC area.

## 3. Experimental Program

### 3.1. Specimen Preparation

**Figure 2:**
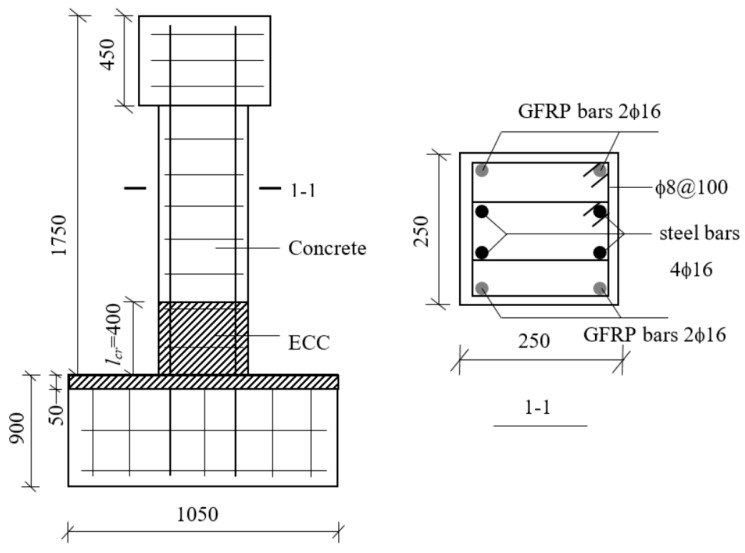
Specimen details (units: mm).

**Figure 3:**
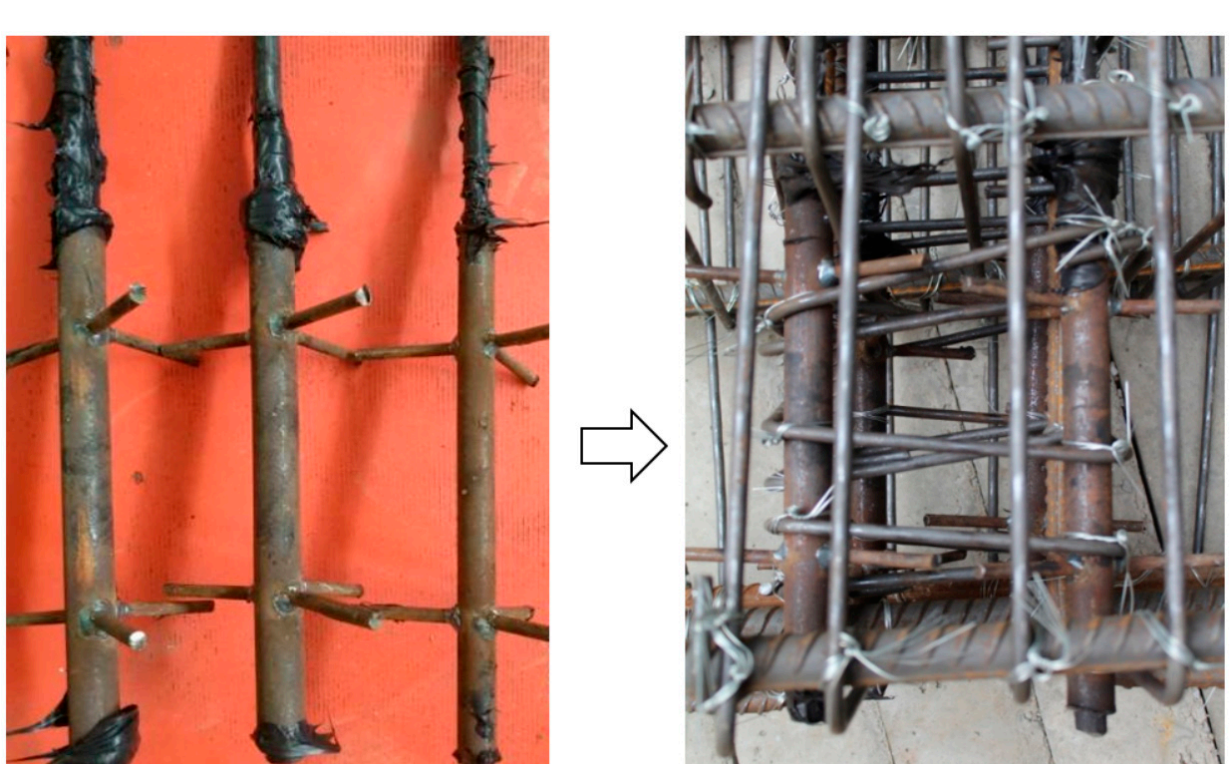
End anchoring of FRP bars.

Two specimens: a hybrid steel—FRP-reinforced concrete (RC) column and a hybrid steel—FRP-reinforced concrete—ECC (RC-ECC) composite column, were tested. Both columns exhibit the same cross section of 250 mm × 250 mm, and length of 1750 mm. The columns were connected to a 450 mm × 900 mm × 1050 mm stub, as shown in Figure 2. For the RC-ECC composite column, the ECC pouring height was determined to be 400 mm based on Equations (1) and (2). The ECC was poured into the stub at a depth of 50 mm to prevent the ECC-concrete interface from being on the same level as the column-stub interface. The pouring scheme of the composite column was as follows: first, pour the concrete area; after the concrete was poured for approximately one hour, i.e., when the concrete entered the initial setting stage, pour the ECC segment. The matrix compositions of the ECC and concrete are displayed in Table 1. The fiber is made of PVA (Polyvinyl alcohol) fiber. The specific performance indexes of PVA fiber are shown in Table 2. Because the FRP bars cannot be bent for anchoring as in steel bars, special anchoring measures were taken at the ends. The FRP bars were inserted into the threaded steel sleeves; subsequently, the structural glues were poured to fill the gaps, as shown in Figure 3. Both columns were reinforced with four glass fibre-reinforced polymer (GFRP) bars and four steel bars with the same diameter of 16 mm. The reinforcement ratios of GFRP bars is 1.29%. The GFRP bars were placed at four corners of the column. For both specimens, the sufficient amount of transverse reinforcement with a diameter of 8 mm and a spacing of 100 mm were employed to prevent shear failure. The geometric dimensions and detailed reinforcement layouts of the specimens are shown in Figure 2.

### 3.2. Material Properties

To determine the tensile property of the ECC, three specimens of dimensions 350 mm × 50 mm × 15 mm were prepared and tested. For all specimens, the ultimate strain at the crack localization reached as large as 3%, and the tensile strength exceeded 4.5 MPa. A group of ECCs and concrete cubes of dimensions 100 mm × 100 mm × 100 mm were also prepared and tested under uniaxial compression. Compression strength tests were performed at the age of the column tests. The measured cube strengths (*f_cu_*) of concrete and ECC were 35.8 MPa and 47.3 MPa, respectively. The elastic modulus and tensile strength of the GFRP bars were measured to be 42.3 GPa and 752 MPa, respectively, as shown in Table 3. The material properties of the steel reinforcements with various diameters are shown in Table 4.

### 3.3. Test Setup

**Figure 4:**
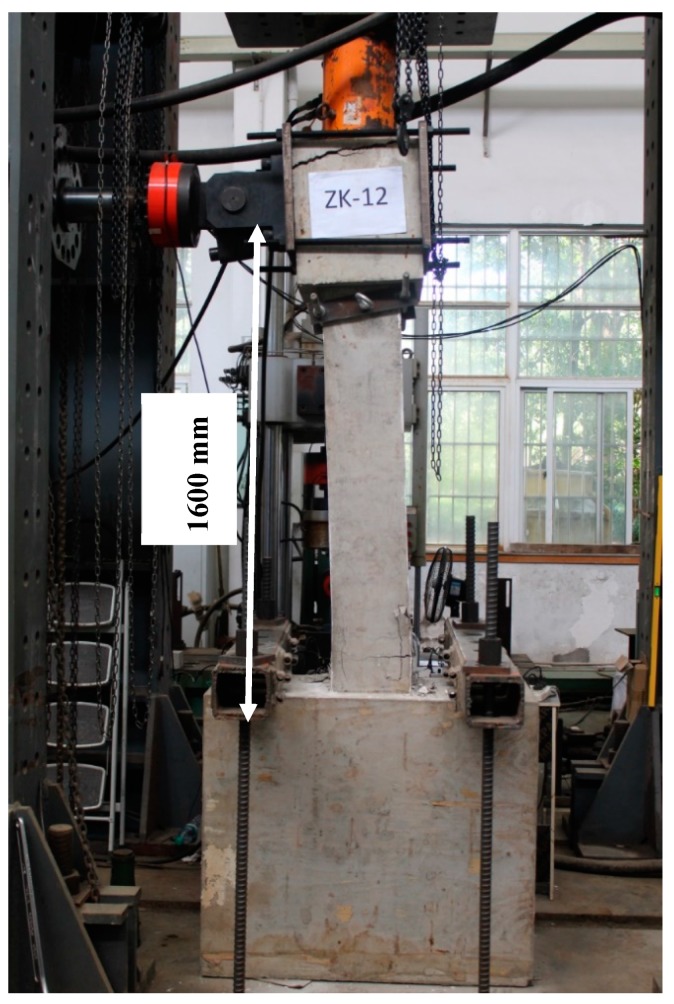
Picture of cyclic loading test.

The columns were tested under a constant axial force and increasing lateral displacements, as shown in Figure 4. The stub was first fixed by two ground anchors. The axial load was subsequently applied at the top of the columns through a hydraulic jack. The axial force ratio was fixed to be 0.3 for both columns, and is defined by the ratio of the applied axial load to the axial load carrying capacity of the columns. Subsequently, a reversed cyclic load was applied via an MTS hydraulic ram affixed on a reaction steel frame. The distance from the centre of the loading to the column base was 1600 mm. The displacement loading scheme at intervals of 2 mm before the lateral displacement of 8 mm and 8 mm afterwards was applied throughout the test. Two cycles were employed at each displacement level. For both columns, the test was terminated when the applied lateral load decreased to 80% of its peak load.

**Figure 5:**
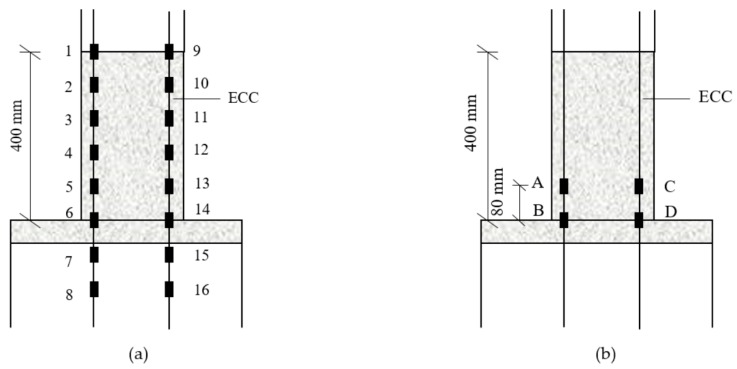
Arrangement of strain gauges on: (**a**) steel bars and (**b**) GFRP bars.

During the loading process, the displacement and load values at the loading point were collected automatically by the data collection system. Additionally, eight strain gauges (120 Ohms) were attached on one of the steel bars at each side at a spacing of 80 mm, and measured during tests. Further, four strain gauges were attached on the GFRP bars to monitor the strain variations. The detailed arrangement of the strain gauges is shown in Figure 5.

## 4. Results and Discussion

### 4.1. Failure Modes and Crack Patterns

**Figure 6:**
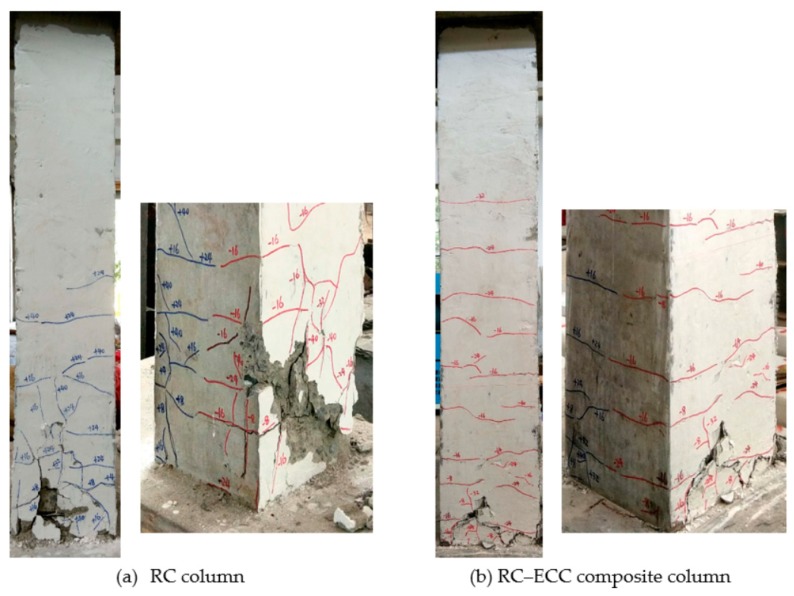
Failure modes and crack patterns of specimens.

The RC column has a shear/span ratio of 6.4, a reinforcement ratio of 2.57%, and an axial force ratio of 0.3. The first flexural crack appeared near the column base at the lateral displacement of 4 mm and the corresponding load of 10.4 kN. With increasing displacement up to 8 mm, the cracks appeared in the range of 200 mm from the column base. At the lateral displacement of 16 mm, flexural cracks developed throughout the entire column section, accompanied by the appearance of longitudinal splitting cracks. The specimen yielded at the displacement of 23.5 mm and the corresponding load of 46.9 kN. Beyond yielding, the lateral resistance remained almost constant with the increase in lateral displacement. At the displacement of 32 mm, significant concrete spalling was observed owing to the reversed cyclic loads. With an evident “bang” sound, the GFRP bars buckled at the displacement of 40 mm owing to the loss of lateral confinement. Immediately after that, the lateral load resistance decreased rapidly to 80% of its peak load and the test was terminated. Finally, the specimen failed by concrete crushing with severe concrete spalling, as shown in Figure 6a. No sign of bond-slip failure of the GFRP bars was observed during the tests, indicating the effective of the anchoring scheme of the GFRP bars for preventing bond failure between the GFRP bars and concrete.

The RC-ECC composite column exhibits the same geometric dimensions and loading configurations as the RC column, while the matrix in the plastic hinge region is replaced by ECCs. The initial tiny crack occurred at the displacement of 8 mm. Subsequently, multiple tiny cracks were observed at the ECC segment. Compared with the RC column, the crack width is smaller and the crack number is significantly greater. The specimen yielded at the displacement of 24 mm at the lateral load of 61.56 kN. With increasing displacement up to 40 mm, flexural crack localised at the column base. Subsequently, new cracks no longer appear while the crack width of the existing cracks continued to develop. The specimen finally failed by ECC crushing at the displacement of 80 mm. However, no evident ECC spalling and GFRP bar buckling was observed during the tests, as shown in Figure 6b. This implies that ECCs can improve the integrity of the specimen, thereby implying a continuous lateral confinement on the GFRP bars. No sign of delamination between the concrete and ECC was observed during the tests. Further, similar to the RC column, no bond failure of the GFRP bars was observed during the entire loading process.

### 4.2. Load Versus Deformation Responses

**Figure 7:**
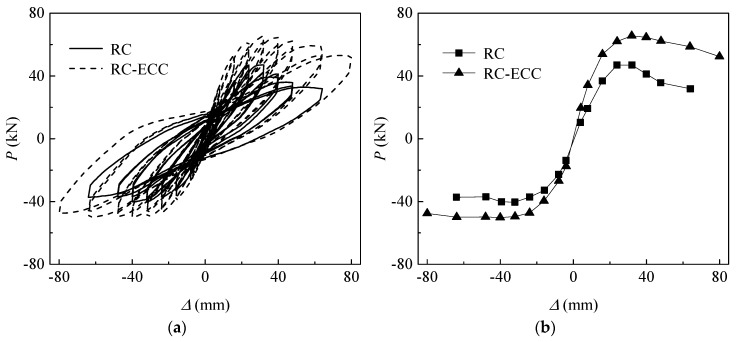
Comparison of cyclic load (*P*) versus lateral displacement (*Δ*) curves between RC and RC–ECC composite columns. (**a**) hysteresis curves; (**b**) envelop curves.

Figure 7a shows the cyclic load (*P*) versus lateral displacement (*Δ*) hysteresis curves of the RC and RC-ECC composite columns. An evident pinch effect was observed for both columns owing to the linear–elastic property of the GFRP bars. At the displacement level of 64 mm, the residual displacements of the RC column and RC-ECC composite column are only 28.96 mm and 18.21 mm, respectively. Figure 7b shows the load versus displacement envelop curves of both columns. As shown, the load carrying capacity of the RC-ECC composite column is much higher (39.62%) than that of the RC column. A higher compressive strength of the ECC compared with that of concrete results in a higher flexural strength of the hybrid-reinforced ECC section. Meanwhile, the tensile resistance of the ECC is equivalent to a higher reinforcement ratio, which also affects the load carrying capacity effectively. Additionally, the ultimate strength and the deformation capacity of the composite column are superior to those of the RC column. The ultimate displacement of the composite column is 80 mm, compared with 45.14 mm of the RC column. The ultimate displacement is defined by the point where the lateral resistance decreases to the 80% of its peak load. For the RC column, a premature local buckling of GFRP bars was observed owing to the severe concrete spalling under reversed cyclic loading. By contrast, no evident ECC spalling was observed, thus providing a stable confinement on the GFRP bars. Consequently, no sign of local buckling of the GFRP bars appeared in the composite column during the tests, resulting in a much higher deformability of the composite column.

### 4.3. Strain Analysis

**Figure 8:**
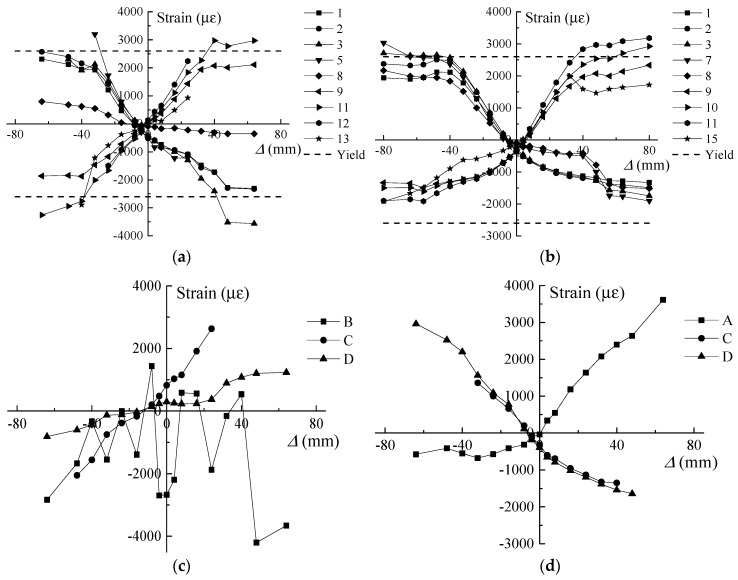
Strain variations of steel and FRP bars with increasing displacement. (**a**) RC, steel bars; (**b**) RC-ECC, steel bars; (**c**) RC, GFRP bars; (**d**) RC-ECC, GFRP bars.

Figure 8 shows the strain variations of the steel and FRP bars of the RC and RC-ECC composite columns at each displacement level. The positive value indicates tension while the negative value represents compression. As shown in Figure 8a,b, the tensile steel strain variations of the RC column are similar to those of the composite column. The strain values in both columns exceed the yield strain of the steel reinforcement. However, when the steel bars are under compression, the steel strain variations are vastly different between these two columns. For the RC-ECC composite column, the steel strains first increase rapidly with increasing lateral displacement, and subsequently remain almost constant below 2000 με when the lateral displacement is larger than 48 mm. However, for the RC column, the steel strains continue increasing up until final failure, and the maximum compressive strain value exceeds 3500 με at the displacement level of 48 mm. This is owing to the significantly concrete spalling at this displacement level, thereby resulting in the compressive stress transfer from the concrete cover to the steel reinforcement. The strain variations of the GFRP bars in the RC and RC-ECC composite columns are also shown in Figure 8c,d, respectively. The maximum compressive strain of the GFRP bars reaches 4201 με in the RC column at the displacement level of 48 mm, indicating local buckling in the GFRP bars. By contrast, the compressive strains of the GFRP bars in the composite column are less than 2000 με during the entire loading process. The significant difference in the compressive strain values in turn causes evident difference in the tensile strain values. The compressive local buckling of GFRP bars in the RC column causes the loss of tensile resistance, whereas the GFRP bars in the composite column provides stable tensile strength throughout the tests, as shown in the tensile strain variations of GFRP bars in Figure 8c,d.

### 4.4. Ductility and Energy Dissipation

**Figure 9:**
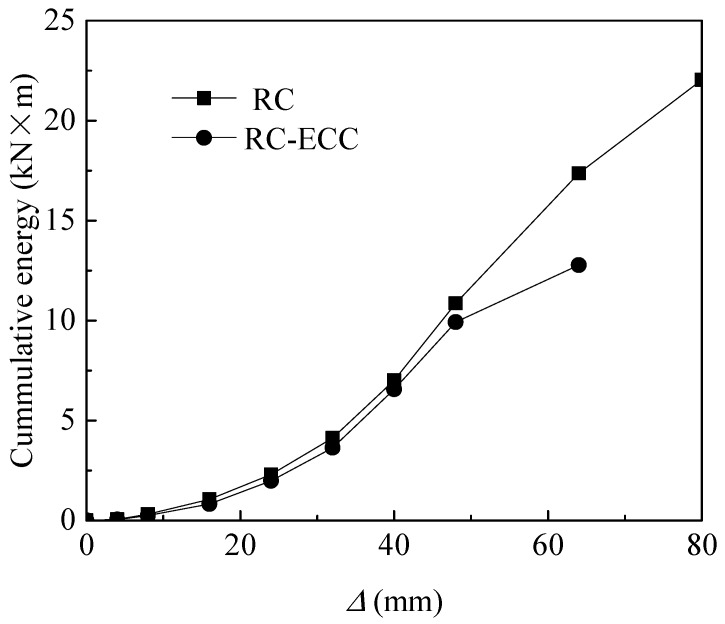
Comparison of cumulative dissipated energy between RC and RC-ECC composite columns.

The ductility index in the present study is defined by the ratio of ultimate displacement to the yield displacement. The ductility index of the RC column is only 1.92, compared with 3.33 for the RC-ECC composite column. The much higher ductility of the composite column compared with that of the RC column is owing to the higher compressive strain at the peak stress, as well as the higher integrity of the ECC compared with that of concrete. The cumulative dissipated energy for both columns at each displacement level are provided, as shown in Figure 9. The dissipated energy at each displacement level is defined by the total area of the load versus displacement loop at each specified displacement. As shown in Figure 9, the cumulative dissipated energy increases rapidly with increasing displacement for both columns. The cumulative dissipated energy of the RC column is slightly lower than that of the RC-ECC composite column before the lateral displacement level of 48 mm owing to the lower lateral resistance of RC column at these periods. When the lateral displacement exceeds 48 mm, the difference in the cumulative dissipated energy becomes increasingly larger. The cumulative dissipated energy of the composite column at the ultimate state (22.04 kN·m) is 72.46% times higher than that of the RC column (12.78 kN·m). This implies that the substitution of ECC with concrete in the plastic hinge zone can improve the energy dissipation capacity of the hybrid steel—FRP-reinforced columns significantly.

## 5. Conclusions

We herein proposed a new type of hybrid steel—FRP-reinforced concrete—ECC composite column with ECC in the plastic hinge zone and concrete in other parts. The mechanical behaviours of the composite column were tested under reversed cyclic loading, which had not been investigated previously. The hybrid steel—FRP-reinforced concrete column was also tested for comparison. The effect of matrix type in the plastic hinge region on the failure model, crack pattern, ultimate strength, ductility, and energy dissipation capacity of the hybrid steel—FRP-reinforced columns was evaluated in detail. The conclusions from the present study are as follows:

1. The failure mode hybrid steel—FRP-reinforced concrete—ECC composite column was different from that of the hybrid steel—FRP-reinforced concrete column. Severe matrix spalling and local buckling of FRP bar were observed in the reinforced concrete column, but did not appear in the composite column. ECC can provide a more stable confinement on the GFRP bars than that of concrete.
2. The anchoring scheme in the present study could present the bond-slip failure between GFRP bars and concrete effectively.
3. With the substitution of ECC with concrete in the plastic hinge region, the load carrying capacity, ductility, and energy dissipation capacity of the hybrid steel—FRP-reinforced column increased by 39.62%, 73.44%, and 72.46%, respectively.

## Figures and Tables

**Table 1:** Mixture proportions.

Matrix Designation	Cement	Fly Ash	Sand	Coarse Aggregate	Water	High-Range Water-Reducing Admixture, %	PVA Fiber Volume Fraction, %
ECC	0.2	0.8	0.2	---	0.22	0.8	2.0
Concrete	1.0	---	1.5	2.5	0.35	0.3	---

**Table 2:** Specific performance indexes of PVA fiber.

Length (mm)	Diameter (µm)	Tensile Strength (MPa)	Elongation (%)	Elastic Modulus (GPa)	Density (g/cm^3^)
12	39	1620	7	42.8	1.3

**Table 3:** Material properties of ECC, concrete and GFRP bars.

Specimen ID	1	2	3	4	5	6	7	8	9	Mean Value	Standard Deviation
Compressive strength of ECC (MPa)	46.0	46.6	46.9	46.4	46.5	46.6	50.1	49.8	47.2	47.3	3%
Compressive strength of concrete (MPa)	37.4	36.8	36.4	34.9	36.7	35.2	35.4	36.9	32.7	35.8	3.82%
Elastic modulus of GFRP bars (MPa)	42	43.5	41.4	--	--	--	--	--	--	--	2.01%
Tensile strength of GFRP bars (MPa)	746	749	761	--	--	--	--	--	--	--	0.86%

**Table 4:** Material properties of steel reinforcement.

Diameter (mm)	Yield Strength fy (MPa)	Ultimate Strength fsu (MPa)	Elasticity Modulus es (GPa)
8	366	524	193
16	528	635	203
